# Safety, Tolerability, and Immunogenicity of COVID-19 Bivalent Vaccination

**DOI:** 10.3390/vaccines11061040

**Published:** 2023-05-30

**Authors:** Divyasha Saxena, Lalit Batra, Shailendra Kumar Verma

**Affiliations:** 1 Center for Predictive Medicine, University of Louisville, Louisville, KY 40202, USA; divyasha.saxena@louisville.edu; 2 Center for Infectious Disease and Vaccine Research, La Jolla Institute for Immunology, San Diego, CA 92130, USA; skverma@lji.org

The COVID-19 pandemic has triggered unparalleled global disruption. However, the development and deployment of effective vaccines provided hope in the fight against the virus. The mRNA-1273 vaccine (Moderna), one of the many COVID-19 vaccines, has shown excellent safety and efficacy towards COVID-19. The vaccine exhibited 93.2% effectiveness against COVID-19 in the Coronavirus Efficacy (COVE) experiment at a median of 5.3 months, following two-dose 100-μg primary serial immunization. This result is a testament to the power of scientific innovation and highlights the importance of ongoing research and development in vaccination [1]. Matkowska-Kocjan et. al. has recently showed the efficacy and immunogenicity of mRNA BNT162b2 (Pfizer/Biontech) vaccine in allogeneic hematopoietic stem cell transplantation (allo-HSCT) recipients, aged 5–11 years, in an outcome of a non-randomized clinical trial in Poland [2]. The success of these vaccines has paved the way for extensive immunization efforts, as well as the prospect of curbing viral spread. As the pandemic continues to evolve, the development of effective interventions, including vaccines, remains a critical priority to work against COVID-19.

The sudden emergence of additional SARS-CoV-2 mutations has been responsible for a significant proportion of COVID-19 cases and deaths. Both Beta (B.1.351) and Delta (B.1.617.2) SARS-CoV-2 viral variants were identified as the most transmissible and capable of evading the immune system response, which is generated by previous infection or vaccination [3]. In 2022, Omicron and its subvariants were identified, with highest antigenic divergence among different SARS-CoV-2 variants [4,5]. Despite establishing population protection through vaccinations or earlier infection, these variations have continued to cause significant sickness and fatalities, underlining the need for new pandemic therapies. To combat the deadly COVID-19 disease, researchers developed a bivalent omicron-containing mRNA-1273.214 vaccine, which is composed of 25 μg each of ancestral Wuhan-Hu-1 and Omicron BA.1 [6]. Chalkias et al. tried to investigate the vaccine’s efficacy and safety in a larger group, and, thereby, a 50-μg bivalent vaccination (mRNA-1273.214) was assessed in relation to a recently enacted 50-μg mRNA-1273 booster. The study looked at the efficacy and safety of the mRNA-1273.214 or mRNA-1273 vaccine in people who had previously been vaccinated with two-dose (100 μg) primary series and one booster shot (50 μg) of mRNA-1273 at least three months prior.

The safety, adverse effects, and potential of the vaccine (mRNA-1273.214) to stimulate an immunological response at day 28 after taking the booster dose were evaluated. During this study, 437 individuals were administered a 50-μg dosage of mRNA-1273.214, and 377 individuals were administered mRNA-1273 as an additional booster. The researchers discovered that those individuals who received the mRNA-1273.214 booster had higher neutralizing antibody levels against the omicron BA.1, BA.4, and BA.5 variants than those who received the mRNA-1273 booster. Furthermore, compared to mRNA-1273 booster, mRNA-1273.214 booster demonstrated more significant neutralization responses against numerous variants, such as Alpha, Beta, Gamma, and Delta. The study did not examine the vaccines’ effectiveness, but, in a preliminary analysis, eleven and nine participants were infected with SARS-CoV-2 after taking mRNA-1273.214 and mRNA-1273 boosters, respectively. This study found that the bivalent vaccine (mRNA-1273.214) produced more effective neutralizing antibody responses than mRNA-1273 vaccine, with no significant differences or problems reported regarding safety.

Researchers also showed the immunogenicity and efficacy of bivalent mRNA vaccines; mRNA-1273.214 (25 μg each of ancestral Wuhan-Hu-1 and Omicron BA.1) and mRNA 1273.222 (Wuhan and Omicron BA.4/5) were shown to be effective against Omicron BA.5 infection in h-ACE2 transgenic mice [7]. The study stated that two-dose immunization with either of the bivalent vaccines induced broadly neutralizing antibody response and improved pathology with minimal inflammation in the lungs compared to the monovalent mRNA-1273 vaccine. However, the authors noted that infection with other SARS-CoV-2 VOCs and higher animal models were required to completely understand the protection and neutralization response.

CDC has already shown the absolute and relative vaccine effectiveness (VE) against symptomatic SARS-CoV-2 infection for a bivalent mRNA COVID-19 booster [8] received after two, three, or four doses of monovalent vaccine (*N* = 360,426) during the BA.4/5 sublineage predominance period (including BA.4.6, BA.5.2.6, BF.7, BQ.1, and BQ.1.1). CDC also reported the vaccine effectiveness of the bivalent mRNA booster against symptomatic XBB/XBB.1.5 infection (*N* = 29,175) in immunocompetent peoples for at least the first three months after bivalent vaccination [9].

In a recently published study [10], Peled et al. evaluated tolerability and immune response profile of the bivalent Omicron-containing vaccine in heart transplant recipients (HTxRx). Serum samples were tested for anti-RBD IgG antibodies, SARS-CoV-2 specific T-cell response, and neutralizing antibodies against Wuhan, Delta, and Omicron variants (BA.1, BA.2, BA.4, and BA.5)

Based on the findings discussed above, bivalent vaccines could play an important role in responding to emerging variants for COVID-19. As compared to the standard, bivalent vaccines produced stronger neutralizing antibody responses without any safety concerns [11]. Wang et. al. already showed the serum neutralization response generated following bivalent vaccination against Omicron sublineages BA.1, BA.2, BA.4–BA.5, BA.4.6, BA.2.75, BA.2.75.2, and several related sarbecoviruses, including SARS-CoV, GD-pangolin, GX-pangolin, and WIV1 [12] by using pseudovirus neutralization assays. Recently, Lippi et. al. designed an epidemiologic model [13] for predicting neutralization against BA.4/5, BA.4.6, BA.2.75.2, BQ.1.1, and XBB.1, based on univariate and multivariate analysis upon FFRNT_50_ values (>50% suppression of fluorescent foci fluorescent focus reduction neutralization test). Limitations of all the above-mentioned studies include non-randomized trial, small sample size, and antibody durability post bivalent vaccine administration. Moreover, bivalent vaccines are not authorized for younger children (from six months to four years of age).

The original mRNA vaccines (Pfizer and Moderna) were based on the Wuhan strain, with restricted administration, despite antibody waning past six months [14]. The main target for bivalent vaccines imprinting is to reduce severe COVID-19 cases against newly emerged SARS-CoV-2 variants, especially for immunocompromised persons. Additional research and clinical trials are required to fully understand the efficacy and safety of bivalent vaccines in response to emerging variants, such as BQ.1 and BQ1.1. and BF.7 subvariants. This editorial welcomes the manuscript submission related to vaccine effectiveness (VE), immunogenicity, and long-term immune response, following bivalent vaccination in normal and immunocompromised individuals. 
